# Touch-free measurement of body temperature using close-up thermography of the ocular surface

**DOI:** 10.1016/j.mex.2016.05.002

**Published:** 2016-05-09

**Authors:** Benjamin Vogel, Heike Wagner, Johanna Gmoser, Anja Wörner, Anna Löschberger, Laura Peters, Anna Frey, Ulrich Hofmann, Stefan Frantz

**Affiliations:** a Comprehensive Heart Failure Center (CHCF), University Clinics of Würzburg, Germany; b Center for Experimental Molecular Medicine, University of Würzburg, Germany; c Department of Internal Medicine I, University Clinics of Würzburg, Germany; d Department of Experimental Biomedicine, University Clinics of Würzburg, Germany; e Universitätsklinik und Poliklinik für Innere Medizin III, University Clinics of Halle (Saale), Halle (Saale), Germany

**Keywords:** Close-up thermal imager for temperature measurement and detail investigations, Thermography, body temperature, ocular surface temperature, myocardial infarction, BT, Body temperature, OST, Ocular surface temperature, rOSTd, relative ocular surface temperature decline, MI, myocardial infarction, LAD, left anterior descending artery

## Abstract

In experimental animal research body temperature (BT) is measured for the objective determination of an animals’ physiological condition. Invasive, probe-based measurements are stressful and can influence experimental outcome. Alternatively BT can be determined touch-free from the emitted heat of the organism at a single spot using infrared thermometers [1]. To get visual confirmation and find more appropriate surfaces for measurement a hand-held thermal imager was equipped with a self-made, cheap, 3D-printable close-up lens system that reproducibly creates eight-time magnified thermal images and improves sensitivity. This setup was used to establish ocular surface temperature (OST), representing the temperature of the brain-heart axis, as a touch-free alternative for measurement of BT in mice, rats, rabbits and humans.OST measurement after isoflurane exposure and myocardial infarction (MI) experiments in mice revealed high physiological relevance and sensitivity, the possibility to discriminate between MI and sham operations in one hour and even long-term outcome-predictive capabilities of OST after MI. Summarized here we present:

• Self-made close-up lens for thermal imaging cameras for eight-time magnification
• Establishment of OST for touch-free determination of BT in rodents and humans
• Short- and long-term predictive capabilities of OST in experimental MI in mice.

## Graphical abstract

**Figure:**
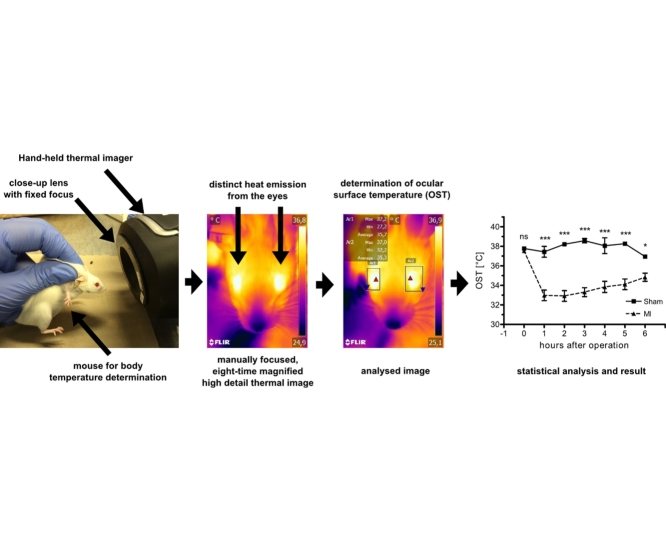

## Method details

Body temperature (BT) is widely accepted and regularly used for objective determination of overall body function. In a broad spectrum of pathologic conditions such as sepsis but also in sterile inflammation BT rises or drops rapidly and represents an important prognostic indicator [2]. In experimental animal research BT is used for physiological surveillance but also to define humane endpoints e.g. to abort an experiment before the animal suffers excessively or dies. Such thresholds have been suggested for several disease models for example in viral or bacterial infection or shock [3], [4], [5]. BT is commonly measured in the rectum since temperature is well preserved in the rectum due to low heat diffusion and removal [6]. However, rapid temperature changes cannot be detected. As an alternative telemetric temperature surveillance by implanted probes was developed [7], [8]. However, this procedure constitutes a considerable burden for the animal, requires a recovery phase and can influence the animal’s physiology and experimental outcome.

To measure temperature non-invasively infrared radiation can be used that is naturally emitted from the surface of every object. Using a hand-held laser-spot guided infrared thermometer the chest surface temperature from mice was measured and correlated with rectal BT [11]. However this fast and flexible approach that reduces animal fixation time resulted in too low, non-physiological temperatures, probably due to the isolating fur at the measuring spot. Furthermore, analysis of a single defined spot (here: xiphoid process) on the chest surface seems questionable for reproducible temperature measurements, since it can be strongly influenced by the isolating fur and heat accumulation under the animal. Additionally it is unclear from the method description how a reproducible distance between thermometer and object is kept constant and how this can influence measurement results. This is crucial since thermal radiation is known to be influenced by environmental factors.

**Fig. 1:**
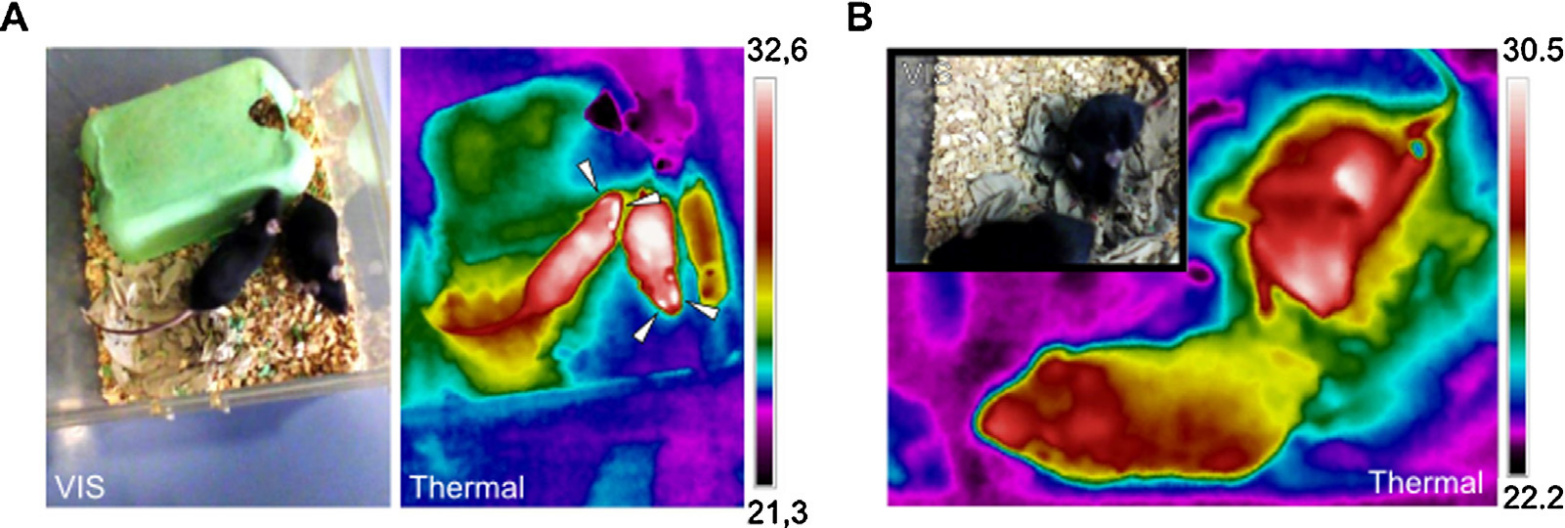
Conventional thermal imaging of mice in cage. A: Demonstrated are visual (VIS) and thermal images (thermal). Note that thermal radiation is reflected and cannot pass through the transparent cage. Note that the eyes of the mice distinctly stand out from the body surface (indicated with arrows). B: Thermal and visual images from the same mice as in A with a distance below 0.5 m are presented. In comparison to A the thermal image is unfocused. Tthe maximum temperature in the image, as indicated in the scale, is 2 °C lower, demonstrating loss of thermal information in unfocused images. Color schemes are rainbow high contrast.

Compared to a single spot infrared thermometer, thermography generates images that can be analyzed on a per pixel basis as if thousands of infrared spot thermometers are used simultaneously. Using thermal imaging we were quickly able to identify the eyes as the most prominent and warmest surface on mice (Fig. 1). However the build-in optics did not allow close-up images from the mouse eyes. By using the thermal focus lens with fixed focus distance that is described here, we were able to create are reproducible distance to the object whenever a sharp image was achieved and so improved thermal sensitivity, which allowed quantification.

## Materials and methods

### Thermal-imaging and focus lens for close-up imaging

A factory-calibrated handheld thermal imaging camera (FLIR E8, FLIR Systems, Inc.) with 320 × 240 pixels (9 Hz refresh-rate) and a built-in screen for instant visual focus control was used for all thermal imaging experiments. Built-in thermal optics focuses from about 0.5 m to infinite automatically. The camera cannot acquire focused images at closer distances than 0.5 m. Unfocused images furthermore provide incorrect thermal information.

The thermal imager is also equipped with a co-axial visual light camera. Normally a visual image is taken whenever a thermal image is acquired. However when the close-up lens is attached the visual light camera is covered and a black image acquired. To clearly assign images to an animal after a series of close-up images were acquired, the lens was removed and an image of a sheet of paper with the identification of the animal/timepoint/etc. was acquired. So finally a series of images were interrupted by identification images, which allowed rapid screenings and identifications.

Lens holder was 3D-printed by the Rechenzentrum of the University of Würzburg using black ABS polymer from the files available from thingiverse: http://www.thingiverse.com/thing:187166. A biconvex zinc-selenide (ZnSe) lens (100% ZnSe, diameter: 20 mm, focal distance: 56 mm, EPSYS Invent, Nürnberg) was attached to the 3D-printed lens holder and attached in front of the camera lens by friction without any modifications to the camera for close-up imaging. With the lens attached focused images could only be acquired in 56 mm focus distance to the object. The pixel size of a focused close-up image at 56 mm was determined from an object of known length at 125 μm x 125 μm. The standard optic only achieves a minimum pixel size of 1 mm x 1 mm at a minimum focus distance of ∼500 mm. This corresponds to an eight-time magnification for the focus lens. For all measurements emissivity coefficient in the camera was set to 0.95.

For all experiments the thermal camera was turned on at least 30 min before quantitative image acquisition to warm up the camera sensor unit, which is mandatory for correct quantification. The camera should be connected to the charger for longer experiments to prevent unwanted turning off.

### Acquisition of ocular surface temperature (OST)

**Fig. 2:**
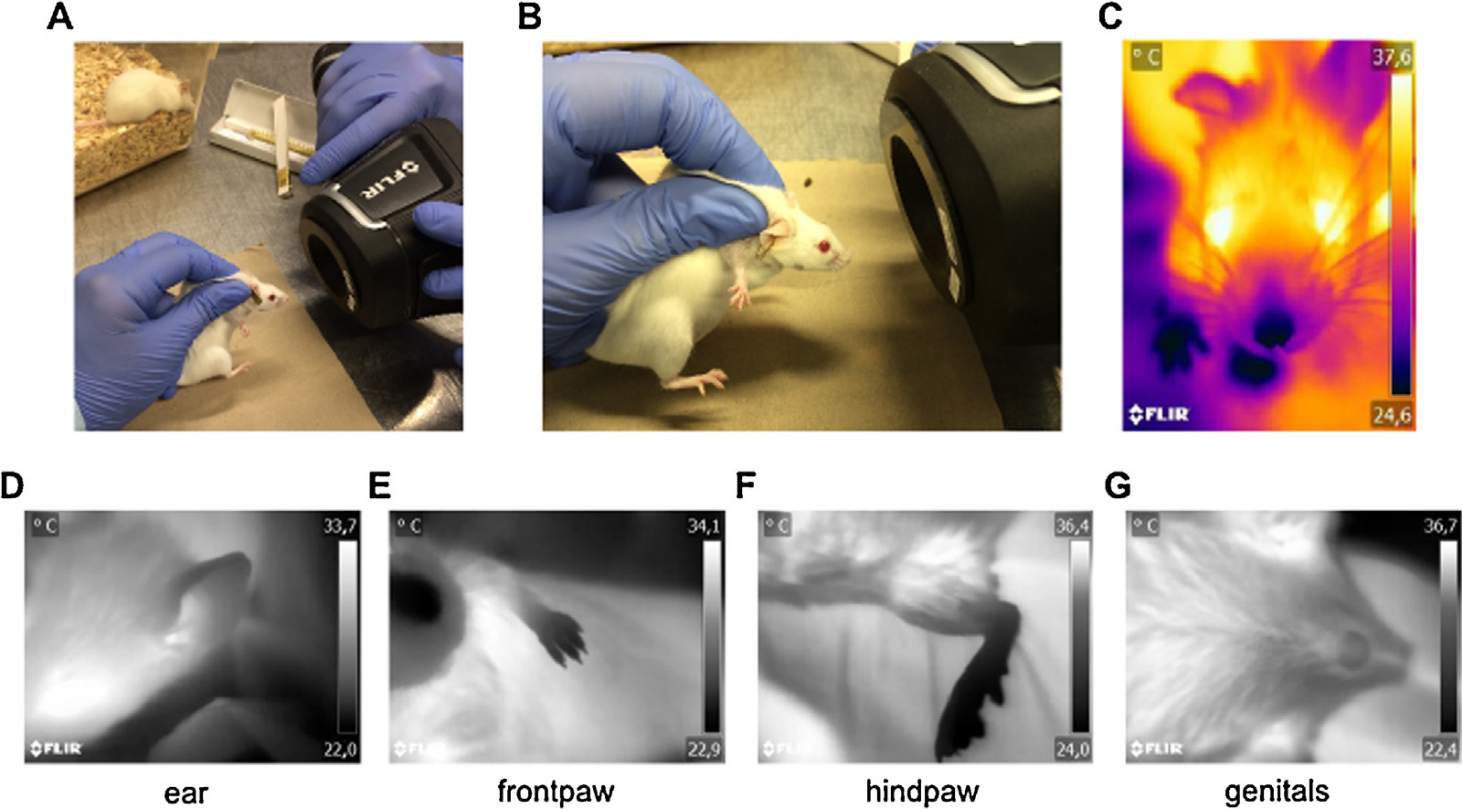
Creating facial close-up images for OST determination. A: A mouse is dorsally fixated by a technician. A second person adjusts the focus by manually moving back and forward the thermal imaging camera with the focus lens attached. B: Detail of the focus distance to the facial area of the mouse for OST acquisition. C: Typical facial image of a mouse for OST determination. Colour scheme is iron. Examples for use of the close-up lens on the mouse body surface. Demonstrated are several close-up images of the healthy murine body: D: ear, E: hindpaw, F: frontpaw, G: genitals. Note that the mouse is fully conscious and alive for imaging. Color scheme is grey.

C57BL/6J or CD-1 mice were dorsally fixed on a stable platform during routine handling in animal husbandry. Another person approached the mouse from the front with the thermal imaging camera and the focus lens attached until a focused facial imaged of the mouse appeared. 3–5 focused facial images were acquired within 15–20 s and analyzed as described below. OST acquisition was performed identically for mice, rats (Wistar) and rabbits (New Zealand White). The thermal imaging camera with the close-lens attached can also be used for detailed thermal investigations of the mouse surface (Fig. 2) and is ideally suited to monitor e.g. the course of inflammatory processes or to detect sick animals amongst others, since they mostly have a lower body temperature.

### OST determination from close-up images (post-processing)

**Fig. 3:**
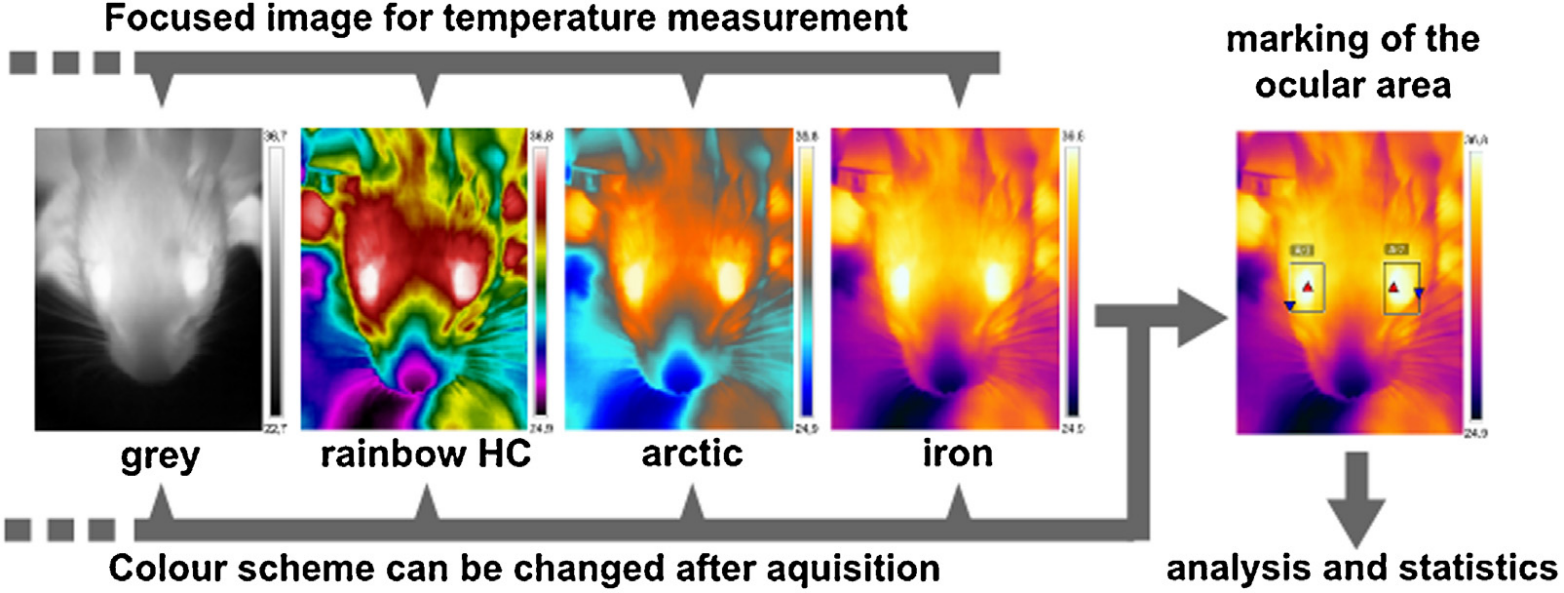
Post-processing for OST determination from focused close-up images. Only focused images are included to analysis. The ocular area is marked with a rectangle. Hottest temperature in this area is indicated with a red triangle. This red triangle must be within the ocular area since this indicates the OST for the area. Otherwise the shape of the rectangle has to be changed until this criterion is fulfilled.

FLIR Tools (FLIR Systems, Inc.) and BFIC (Batch Thermal Images Converter, Dave Spilka) software were used for extraction of OST data from close-up images and changes of colour scheme post acquisition (Fig. 3). A thermal image was loaded and the ocular area, which normally is the warmest area in the image, was marked with a rectangular shape using the corresponding marking tool. The hottest spot (maximum temperature) in the rectangle, which is automatically indicated, must be located within the ocular surface (area between the two eyelids) and was defined as ocular surface temperature (OST). If the hottest spot was not located within the ocular surface, the marking rectangle was altered until this criterion was fulfilled. The value of the hottest spot was transferred to MS Excel. BFIC has a batch procedure for this purpose. OST was determined from 3 to 5 focused images individually for each eye. The OST of a mouse was the mean of all determined OSTs of both eyes, if not stated otherwise.

### Verification of ZnSe lens usability for thermal quantification

An adjustable and heatable mouse handling table (Visual Sonics) was used to compare the quantification capabilities of the thermal imaging camera with or without the focus lens attached. A piece of black PVC tape (normally used for electric insulation) was placed on the heating plate and used as a provisional black radiator for unbiased surface measurement. Indicated temperatures were set on the handling table. Two thermal images each with or without the lens attached to the camera were acquired at least 30 s after the desired temperature was reached on the controller display. Thermal images without lens were acquired in closest possible proximity to yield a sharp image (∼500 mm). Thermal images with the lens attached were acquired in focus range (focus on the edges of the PVC tape, 56 mm). For temperature determination on the black PVC tape in the images the area was marked as described above for OST analysis. The hottest spot on the PVC tape was used for temperature analysis.

### OST after exposure to different isoflurane concentrations

200 μl isoflurane (CP Pharma, Burgdorf, Germany) were pipetted to a cellulose towel in a 1000 ml beaker and evaporated for routine ear-tagging in the animal husbandry. A plastic lid was placed on top of the beaker to prevent isoflurane loss during incubation periods. OST was determined before exposing a mouse to isoflurane. During exposure the mouse was observed and removed after complete relaxation (about 60 s). OST was measured again, before the mouse was ear-tagged. Another two mice were anesthetized in the same beaker successively until a new beaker with fresh isoflurane was used by protocol (n = 3 × 6). Consequently the isoflurane concentration in the beaker reduced gradually after each isoflurane exposure (1st, 2nd, 3rd) due to inhalation, diffusion and handling to the same extent. Notably time until complete relaxation of each mouse was 60 s, even when the mice were exposed to the lowest isoflurane concentration in the beaker.

### Myocardial infarction (MI) and echocardiography

8-weeks old wild-type C57BL/6J mice (Harlan-Winkelmann) underwent permanent ligation of the left anterior descending coronary artery (LAD) as described previously [9]. Briefly mice were intubated and anesthetized with 2 Vol% isoflurane. After shaving, thoracotomy, and opening of the pericardium a ligature of the LAD was set in the beating heart (not in sham animals) to induce MI. All mice received 1.2 μg Buprenovet (0.1 mg/kg) as preemptive analgesia. OST was determined before operation and at indicated time points starting 1 h after operation to avoid confounding effects of isoflurane anesthesia. Echocardiography was performed 3 and 8 weeks after onset of MI by an experienced technician using a Vevo 1100 system (Visual Sonics). After 8 weeks mice were sacrificed, hearts and lungs were excised and weighed [18]. Infarcts were verified by echocardiography and organ weights. The investigation conforms to the Guide for the Care and Use of Laboratory Animals published by the US National Institutes of Health. All experimental procedures were approved by the local government (69/12).

### Conventional BT measurement

For rectal BT measurement a Thermalert TH 5 digital thermometer with a RET-3 probe for mice and a RET-2 probe for rats was used (Physitemp). For rectal BT measurement in rabbits a standard digital thermometer was used (Eco temp basic, Omron). One rectal measurement took about one minute until the inserted thermometer showed an equilibrated temperature.

### Human ear temperature and OST acquisition

BT of 33 healthy volunteers (informed written consent, male and females, age: 18–65) was determined three times per ear using an infrared ear thermometer (Braun/Welch-Allyn, Thermoscan Pro 4000). Then three focused close-up images per eye were acquired for OST analysis and further analyzed as described above. All data were handled anonymously. This human study was approved by the ethics committee of the University of Würzburg.

### Statistical analysis

Graphpad Prism 4.0 was used for statistical analysis. Student’s *t*-test was used for comparison of two groups. Multiple groups were analyzed by one-way ANOVA and indicated post-hoc tests. Correlation analysis was done using Pearson’s correlation. Two groups were considered significantly different with p < 0.05 (*), p < 0.01 (**) and p < 0.001 (***), otherwise not significant (ns). All indicated values are means ± standard error mean (SEM).

## Results and discussion

### Verifying thermal information using the focus lens

To verify that close-up imaging still provides correct thermal information a digitally controlled heating plate and corresponding surface temperatures were measured. An almost perfect correlation between set and determined temperatures with (r = 0.9991) or without lens (r = 0.9983) was observed (Pearson, n = 17). We concluded that the focusing lens does not alter absolute thermal information in the range from 34 to 50 °C and can also be used for quantification purposes.

### OST measurement delivers physiologically relevant temperatures

Next, we wanted to investigate if close-up thermal imaging of the mouse eye results in physiologically relevant OSTs close to rectal values (37.0–39.0 °C) [10]. Parallel measurements of OST and rectal BT revealed no significant differences between both locations (rectal BT vs OST: 37.5 ± 0.2 °C vs 37.2 ± 0.2 °C, p = 0.1206, n = 16, two-tailed *t*-test).

To investigate the precision of OST measurements the means of the standard deviations (SD) and standard errors (SE) from 56 analyzed animals (3–5 images each) were calculated. The mean SDs (0.3 ± 0.1 °C) and mean SEs (0.1 ± 0.1 °C) were within the camera’s detection sensitivity range (0.1 °C). OST from the left or right eye were found to be not significantly different (left vs right: 36.9 ± 0.1 °C vs 36.9 ± 0.1 °C, n = 25, p = 0.8416, two-tailed *t*-test), thus both eyes can be used for OST determination.

To rule out that dorsal fixation for OST acquisition creates stress to the animal that influences OST, mice were fixed dorsally, images for OST acquired, the mouse released and immediately fixed again for another OST acquisition. Comparison of OSTs revealed no significant differences before and after handling, which suggest that handling does not affect OST (before vs after: 36.8 ± 0.2 °C vs 36.5 ± 0.3 °C, n = 8, p = 0.2759, two-tailed *t*-test). Together, these results suggest that OST acquired by close-up thermal imaging is an alternative to rectal BT determination. OST is reliable, delivers physiological temperatures with minimal variations, and can be readily measured from both eyes independently.

### Rapid OST reaction to external stimuli

**Fig. 4:**
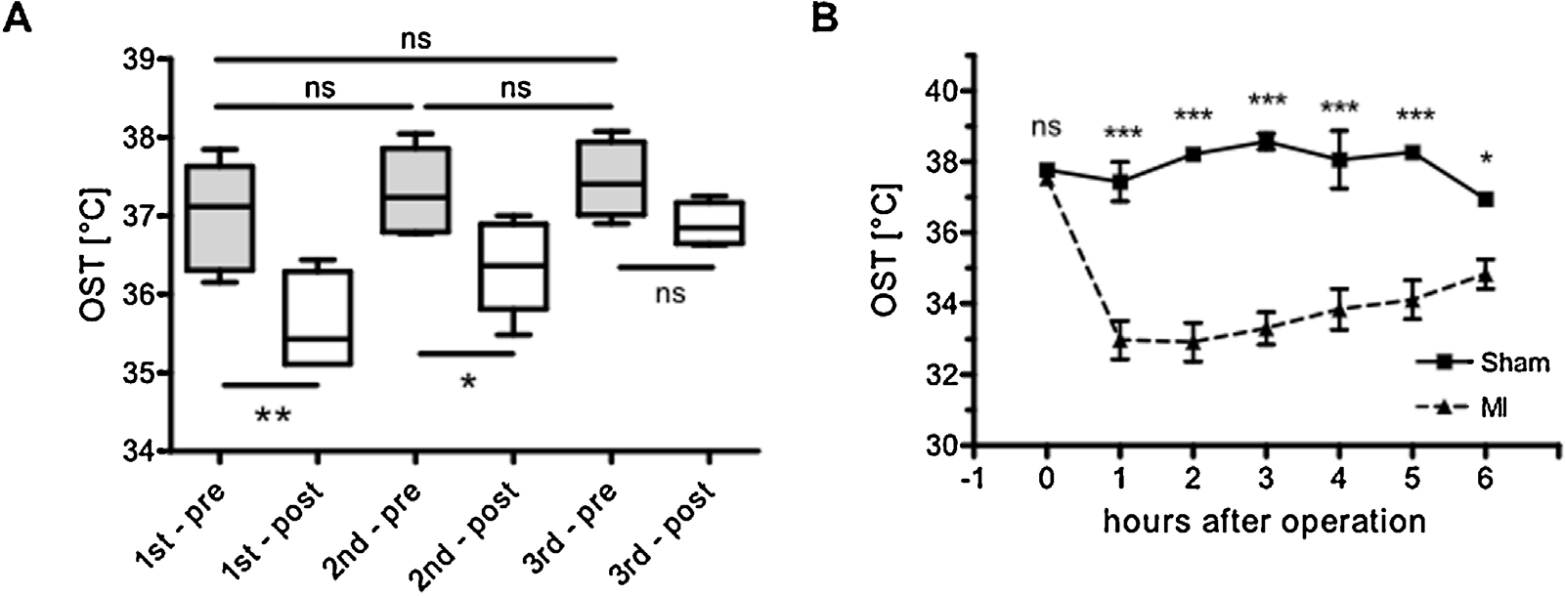
Measurement of OST after isoflurane exposure and experimental MI. A: Box plot of OSTs determined before (pre, grey boxes) and after (post, white boxes) incubation with steadily in 3 steps decreasing isoflurane concentrations [1st, 2nd, 3rd; n(each) = 6]. No differences in EST before incubation were detected [1st – pre: 37.0 ± 0.3 °C; 2nd – pre: 37.3 ± 0.2 °C; 3rd – pre: 37.5 ± 0.2 °C; Bonferroni post-test. After incubation OST dropped in a concentration dependent manner [1st – post: 35.6 ± 0.2 °C, 2nd – post: 36.4 ± 0.2 °C, 3rd – post: 36.9 ± 0.1 °C, one-way ANOVA, Bonferroni post-test] p values are as indicated. B: OST is declining after MI but not after sham operation: Sham (0 h): 37.8 ± 0.1 °C, sham (1 h): 37.4 ± 0.6 °C, sham (2 h): 38.2 ± 0.2 °C, sham (3 h): 38.6 ± 0.2 °C, sham (4 h): 38.1 ± 0.8 °C, sham (5 h): 38.3 ± 0.2 °C. MI (0 h): 37.5 ± 0.1 °C, MI (1 h): 33.0 ± 0.5 °C, MI (2 h): 32.9 ± 0.6 °C, MI (3 h): 33.3 ± 0.5 °C, MI (4 h): 33.8 ± 0.6 °C, MI (5 h): 34.1 ± 0.6 °C, two-way ANOVA, repeated measurements, n(sham vs MI) = 8 vs 16, p values are as indicated.

We wanted to investigate if OST measurement is sensitive enough to monitor small and rapid changes in body temperature. For this purpose mice were exposed to three descending concentrations of isoflurane [1st, 2nd, 3rd; n(each) = 6], which is known to reduce body temperature in a concentration dependent manner [11]. No differences in OST were detected in all groups before isoflurane exposure. However, after isoflurane anesthesia, OST dropped in a concentration dependent manner (Fig. 4A). The first group of mice encountered a robust OST drop of 1.4 ± 0.1 °C, the second group a drop of 0.9 ± 0.2 °C and the third group a drop of 0.6 ± 0.1 °C. A strong linear trend between OST drop and decline of isoflurane concentration could be found (repeated measures ANOVA, post-test for linear trend, p < 0.001). This experiment demonstrates that even small thermo-depressive effects by isoflurane exposure in mice can be rapidly determined by OST measurement.

### OST drops after induction of myocardial infarction but not after sham operation

OST measurement was then applied in a murine model of myocardial infarction (MI). Ischemia and necrosis of cardiomyocytes in the heart lead to acute and long-term changes in cardiac function measurable by echocardiography (Fig. S1). Notably within 1 h after MI surgery OST dropped significantly compared to baseline OST. In some animals OST decreased for more than 8 °C with a steady increase during the following hours (Fig. S2). In contrast, mice undergoing sham surgery demonstrated no significant changes in OST up to 5 h after operation compared to baseline conditions. Comparison of serially measured OST in sham and MI mice revealed a significant difference between groups from 1 to 6 h after MI (two-way ANOVA, repeated measurements, Fig. 4B). For better comparability the relative OST decline (rOSTd) was introduced, which is individually calculated for each animal from the OST before MI (0 h) vs the OST at a specified time point t [OST (pre MI)- OST (post MI)]/OST (pre MI) x 100%]. Non-surviving animals were excluded from rOSTd analysis (MI vs sham n = 12 vs 10). In the MI group rOSTd was 10.8 ± 2.0% (1 h), 8.7 ± 2.0% (2 h), 6.9 ± 1.9% (3 h), 6.9 ± 1.5% (4 h), 8.9 ± 1.1% (5 h), 5.4 ± 1.1% (6 h). In the sham group rOST was 2.9 ± 1.5% (1 h), −0.4 ± 0.5% (2 h), −1.1 ± 0.4% (3 h), −1.5 ± 0.8% (4 h), 1.6 ± 1.4% (5 h), 2.0 ± 0.5% (6 h). This makes clear that acutely decreasing OST did not reflect anesthesia or thoracotomy but implies a particular connection to myocardial injury after MI. OST could be used to effectively discriminate correctly-induced experimental MI from sham or technically- wrong operated animals within one hour after operation. Echocardiography allows this discrimination not earlier than 24 h after operation. This technique might be useful for experiments were a certain amount of correctly operated animals are absolutely necessary. So more animals can be operated when it gets clear that technical problems during operation occurred that will not lead to a correctly infarcted animal.

### Acute OST decline after MI correlates with cardiac dysfunction in the long term

In the long-term MI leads to deteriorated heart function and congestive heart failure with increased lung weight as a marker (Fig. S3). Echocardiographic parameters from 21 and 56 days after MI were correlated with rOSTd at 4, 5 and 6 h after operation of the MI group to investigate connections between acute OST decline after MI and cardiac function in the long term (Fig. S4). Strong Pearson correlations of rOSTd at 5 and 6 h, but not 1–4 h after MI with typical echocardiographic parameters of left ventricular dilation and systolic function were detected (Fig. S4, n = 12). For example rOSTd 6 h after MI strongly correlated with end diastolic areas of the left ventricle at the midpapillary level [PA EDA: r(21d) = 0.7152, r(56d) = 0.7418, Fig. S4] and 2D fractional shortening [2D PA FS (21d and 56d): r(21d) = −0.6444, r(56d) = −0.6748, Fig. S4] determined both 21 and 56 days after MI. Left ventricular to body weight ratios also showed a strong correlation with rOSTd 6 h after MI (r = 0.7429, Fig. S4), while lung to body weight ratios only weakly correlated (r = 0.4817, Fig. S4). These results indicate a direct relationship between rOSTd, reflecting acute cardiac injury in MI, with hypertrophy and long-term cardiac dysfunction. This suggests that rOSTd 6 h after MI in mice predicts long-term cardiac outcome, allows efficient discrimination between sham and successful MI surgery and therefore may present a useful tool in cardiovascular science in the future.

### Measurement of OST in rats, rabbits and humans

**Fig. 5:**
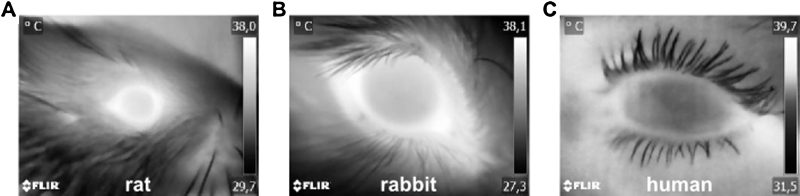
Representative thermal close-up images from eyes of rats, rabbits and humans. A: rat, B: rabbit, C: human. Note that in rats and rabbits the brightest area in the image is the eye surface, while in the image from the human eye warmer areas are visible outside the ocular area and the human eye also is colder in the center. In rodents the eye areas emit hottest radiation compared to the rest of the animal due to the isolating fur. That isolating fur does not exist in humans, therefore heat also emits everywhere else. However exactly the same criteria for OST determination were applied for humans and rodents from the reproducibly detectable ocular area. Color scheme is grey.

It was investigated if OST can also be measured and applied in other organisms than mice. For this purpose OST was measured together with an established method as rectal measurement (rat, rabbit) or infrared ear thermometer (human) under basal conditions and compared. In rats (basal rat rectal BT: 36.0–40.0 °C, [10]) OST was significantly higher than rectal BT(rat rectal vs OST: 35.7 ± 0.1 °C vs 36.5 ± 0.2 °C, n = 15, two-tailed *t*-test, p < 0.001, Fig. 5A). The same was observed in rabbits (basal rabbit rectal BT: 38.5–40.0 °C, [10], rabbit rectal vs OST: 38.2 ± 0.2 °C vs 39.1 ± 0.2 °C, n = 18, two-tailed *t*-test, p < 0.01; Fig. 5B). In humans OST and BT measured from the ear were both in the physiological range (basal human rectal BT: ∼36.5–37.5 °C) with OST being slightly higher, but not significantly different (human ear vs OST: 36.9 ± 0.1 °C vs 37.1 ± 0.1 °C, n = 33, two-tailed *t*-test, p = 0.4738, Fig. 5C). It has to be noted that the human facial area does not contain any isolating fur that covers the area around the eye. Therefore the human eye was not as distinctly different in its temperature from the surrounding tissue. However the OST from the human eye was analyzed exactly as demonstrated in all rodents. Together the data demonstrate that OST measurement delivers physiologically relevant values in all organisms examined here. Furthermore, there is a significant temperature difference depending on the location of measurement, with a higher temperature at the head than at the rectum. Thus, OST is likely to reflect brain temperature or generally the cephalo-thoracic axis, that is known to be higher than in the abdomen [6].

### Additional information

Using thermal imaging we easily identified the eyes as the warmest surface on the body surface of mice. Since the eyes are supplied by blood from the ophthalmic artery from, the brain, we thought that the ocular surface temperature (OST) might be a non-invasive approach to measure the temperature of the highly important brain-heart axis in mice by close-up thermography. This is supported by the fact that the cephalo-thoracic region maintains the highest BT, as described in pigs using thermal imaging in a model of acute lung injury [12]. Thus OST appears most appropriate for rapid measurement of core BT even under changing circulatory conditions. From a temperature point-of-view the heart can be considered as a thermal mixing chamber [6]. During acute MI affected cells of the cardiac muscle undergo necrosis and are inevitably lost which affects heart function instantly [13]. Obviously this damage to the thermal mixing chamber, the heart, affects temperature in the eye as we could demonstrate here and allowed discrimination between MI and sham operation within one hour after operation. Furthermore in the long-term the heart undergoes left ventricular remodeling characterized by hypertrophy and dilation [14]. This is characterized by worsened cardiac function detectable e.g. by echocardiography [13], [15].

Mice have an exceptionally high cardiac output per body mass, which is two to ten times higher than in rats or humans [16]. This fact probably results in very quick and effective heat convection from core to periphery. This would not only explain the rapid thermal reactions of mice to isoflurane induced vasodilation but also BT decline after MI. In a mouse model of temporary coronary ligature, cardiac output has been shown to decrease by over 40% already at 45 min after onset of ischemia [15], [17]. Therefore it is not surprising that heat convection from the body core to the head cannot keep pace with heat loss under such acutely impaired perfusion conditions during the hours following MI. Our data suggest that the more OST drops after MI, the more severe must be the acute impairment of cardiac output, and the worse is long-term prognosis for cardiac function. In view of the preliminary, not-demonstrated OST data it might also be possible to predict the extent of heart failure and probability of death after MI on the basis of temperature decline.

Thermal imaging is an underrepresented technology in life science and experimental animal research. The approach presented here strongly improves visual and quantitative data yield in a cost effective manner and will represent a helpful tool for many researchers not only in experimental or cardiovascular animal science. Furthermore, the approach is in full accordance with the world-wide guidelines of the 3Rs (replacement, reduction, refinement) for experimental animal research as it reduces stress and potential injury to the animal during temperature surveillance, adds further valuable visual information and therefore should be applied whenever possible [18].
