# Supplementary material for: Touch-free measurement of body temperature using close-up thermography of the ocular surface
Source: MethodsX. 2016 May 9;3:407–16. doi: 10.1016/j.mex.2016.05.002 (PMC4887592; doi:10.1016/j.mex.2016.05.002)
Supplement: Supplementary file 1 [file mmc1.pdf]

Fig S1

21d		PA ESA	PA EDA	AP ESA	AP EDA	PA EDD	PA ESD	PA EDVW	PA EDPW	AP EDD	AP ESD	AP EDVW	AP EDPW	PA 2D FS	AP 2D FS	PA MM FS	AP MM FS
	**n**	12	12	12	12	12	12	12	12	12	12	12	12	12	12	12	12
**MI**	**Mean**	18,66	21,66	18,46	21,41	0,5464	0,4858	0,05194	0,06278	0,5492	0,4917	0,05056	0,0625	21,98	21,68	13,34	12,88
	**SEM**	2,923	2,513	2,903	2,574	0,03196	0,04334	0,004577	0,005683	0,03083	0,04474	0,004693	0,006681	7,283	7,066	3,824	4,252
	**n**	10	10	10	10	10	10	10	10	10	10	10	10	10	10	10	10
**Sham**	**Mean**	3,151	7,934	3,099	7,74	0,3597	0,2267	0,072	0,08767	0,3607	0,2313	0,07	0,08467	60,89	61,56	37,27	36,96
	**SEM**	0,423	0,3774	0,4469	0,4042	0,00726	0,01328	0,002639	0,001863	0,01287	0,02044	0,003514	0,001805	3,962	4,248	2,791	4,28
	**p (MI vs Sham)**	<0.001	<0.001	<0.001	<0.001	<0.001	<0.001	<0.01	<0.001	<0.001	<0.001	<0.001	<0.001	<0.001	<0.001	<0.001	<0.001

56d		PA ESA	PA EDA	AP ESA	AP EDA	PA EDD	PA ESD	PA EDVW	PA EDPW	AP EDD	AP ESD	AP EDVW	AP EDPW	PA 2D FS	AP 2D FS	PA MM FS	AP MM FS
	**n**	12	12	12	12	12	12	12	12	12	12	12	12	12	12	12	12
**MI**	**Mean**	23,62	26,74	23,16	26,92	0,57	0,5431	0,04167	0,05194	0,6058	0,7178	0,04167	0,05111	17,2	19,17	11,04	11,97
	**SEM**	3,468	3,126	3,519	3,335	0,05311	0,0439	0,003658	0,006685	0,03336	0,1748	0,004723	0,006132	5,066	4,547	2,796	3,006
	**n**	10	10	10	10	10	10	10	10	10	10	10	10	10	10	10	10
**Sham**	**Mean**	4,459	9,552	4,571	9,562	0,3687	0,249	0,06733	0,08233	0,3883	0,3477	0,06367	0,079	53,68	52,9	32,53	31,47
	**SEM**	0,368	0,4334	0,4972	0,4187	0,007083	0,008389	0,003135	0,001795	0,01314	0,08623	0,002742	0,002386	2,313	3,457	1,467	2,35
	**p (MI vs Sham)**	<0.001	<0.001	<0.001	<0.001	<0.01	<0.001	<0.01	<0.001	<0.001	ns	<0.001	<0.001	<0.001	<0.001	<0.001	<0.001

Fig S1: Echocardiographic analysis of left ventricular function 21 and 56 days after myocardial infarction. Abbreviations: PA: papillary, AP: apical, ESA: endsystolic area [mm^2], EDA: enddiastolic area [mm^2], EDD: enddiastolic diameter [cm], ESD: endsystolic diameter [cm], EDAW: enddiastolic anterior wall [cm], EDPW: enddiastolic posterior wall [cm], MM: M-Mode, FS: fractional shortening. For comparison of MI vs Sham student's t-test was performed. Note that there is an ongoing remodeling process from day 21 to day 56 characteristic for congestive heart failure leading to left ventricular dilation and subsequent deteriorated heart function.

Fig S2

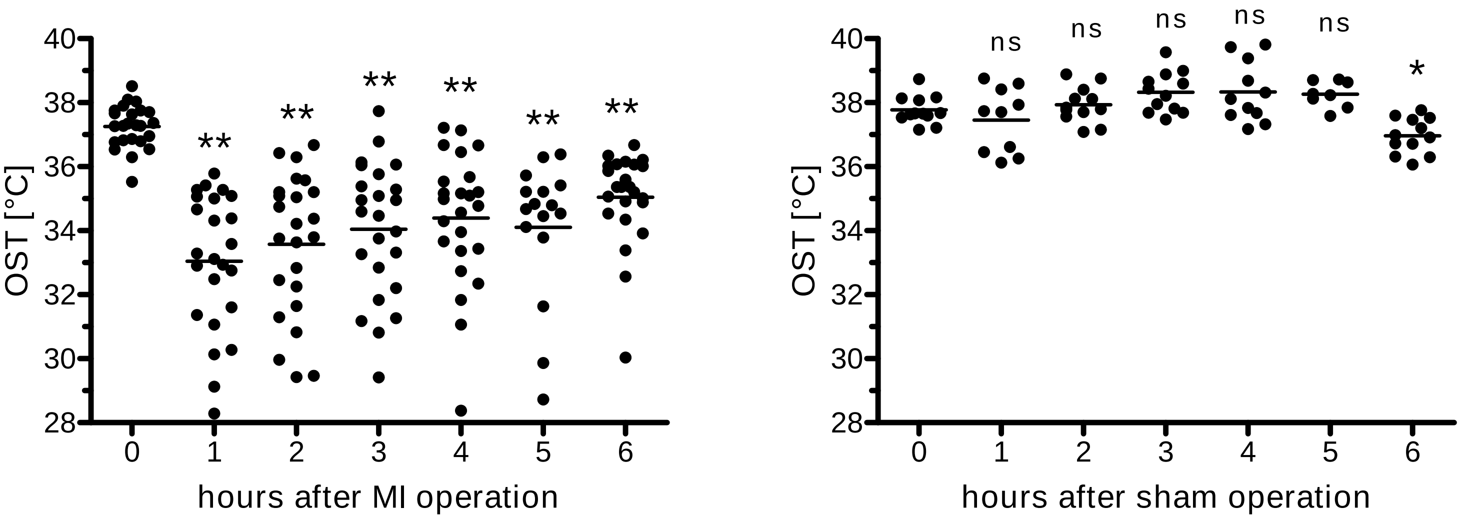

Fig S2. Raw OSTs after induction of myocardial infarction (MI) or sham operation. Left: MI operation, pre-MI (0h): 37.3 ± 0.14 °C, n=24; MI (1h): 33.1 ± 0.44 °C, n=24, p<0.01; MI (2h): 33.6 ± 0.45 °C, n=24, p<0.01; MI (3h): 34.1 ± 0.43 °C, n=24, p<0.01; MI (4h): 34.4 ± 0.42 °C, n=24, p<0.01; MI (5h): 34.1 ± 0.55 °C, n=16, p<0.01, (6h): 35.0 ± 0.30 °C, n=24, p<0.01, one-way ANOVA, Dunnett's post-test (all vs 0h). Right: sham operation, pre-sham (0h): 37.8 ± 0.13 °C, n=12, p=ns; sham (1h): 37.5 ± 0.32 °C, n=10, p=ns; sham (2h): 37.9 ± 0.16 °C , n=12, p=ns; sham (3h): 38.3 ± 0.18 °C , n=12, p=ns; sham (4h): 38.3 ± 0.29 °C , n=11, p=ns, sham (5h): 38.3 ± 0.15 °C, n=8, p=ns, sham (6h): 37.0 ± 0.16 °C, n=12, p<0.05, one-way ANOVA, Dunnett's post-test (all vs 0h)]. Note that the OST of sham animals is relatively constant while the OST of infarcted animals drops after induction of MI

Fig S3

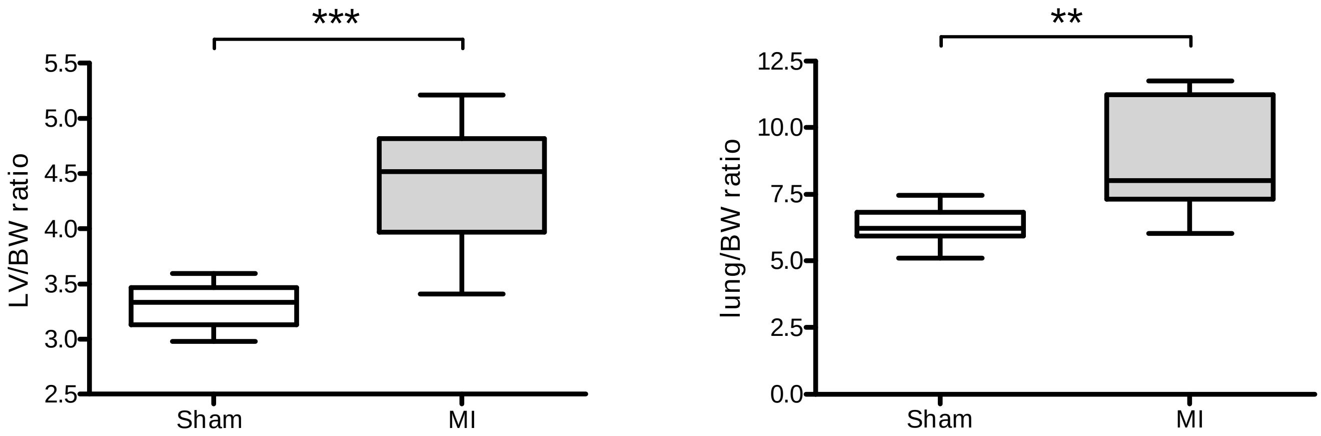

Fig S5. Myocardial infarction in mice leads to left ventricular (LV) hypertrophy and increased lung weight. Left: Comparison of left ventricle weight normalized to body weight (LV/BW) 56 days after sham operation (3.315 ± 0.061) or myocardial infarction (4.429 ± 0.165, p<0.0001, two-tailed t-test, n=10 vs 11). Right: Comparison of lung weight normalized to body weight (lung/BW) 56 days after sham operation (6.296 ± 0.203) or myocardial infarction (8.856 ± 0.660, p<0.002, two-tailed t-test, n=10 vs 11). Note that LV/BW and lung/BW are both significantly increased after myocardial infarction, which is indicating congestive heart failure.

Fig S4

Echocardiography 21d and 4h rOSTd																
Parameter	**PA ESA**	**PA EDA**	**AP ESA**	**AP EDA**	**PA EDD**	**PA ESD**	**PA EDAW**	**PA EDPW**	**AP EDD**	**AP ESD**	**AP EDAW**	**AP EDPW**	**PA 2D FS**	**AP 2D FS**	**PA MM FS**	**AP MM FS**
Number of XY Pairs	12	12	12	12	12	12	12	12	12	12	12	12	12	12	12	12
Pearson r	0,3015	0,298	0,3063	0,2839	0,3359	0,3596	-0,366	-0,4652	0,3455	0,3567	-0,3996	-0,4913	-0,491	-0,5214	-0,4814	-0,4579
P value (two-tailed)	0,3409	0,3468	0,3328	0,3712	0,2858	0,251	0,2419	0,1275	0,2714	0,255	0,1981	0,1048	0,1051	0,0821	0,113	0,1344
R squared	0,09091	0,08883	0,09384	0,0806	0,1128	0,1293	0,134	0,2165	0,1194	0,1272	0,1597	0,2414	0,241	0,2719	0,2318	0,2097
**Echocardiography 21d and 5h rOSTd**																
Number of XY Pairs	12	12	12	12	12	12	12	12	12	12	12	12	12	12	12	12
Pearson r	0,6956	0,7018	0,6859	0,7079	0,5049	0,4733	-0,2039	-0,3806	0,5178	0,5156	-0,2786	-0,3604	-0,5374	-0,5016	-0,4112	-0,484
P value (two-tailed)	0,012	0,011	0,0138	0,01	0,0941	0,1202	0,525	0,2223	0,0846	0,0862	0,3805	0,2497	0,0715	0,0966	0,1842	0,1109
R squared	0,4839	0,4925	0,4704	0,5011	0,2549	0,224	0,04157	0,1449	0,2682	0,2659	0,07763	0,1299	0,2888	0,2516	0,1691	0,2343
**Echocardiography 21d and 6h rOSTd**																
Number of XY Pairs	12	12	12	12	12	12	12	12	12	12	12	12	12	12	12	12
Pearson r	0,7149	0,7152	0,7272	0,7264	0,6809	0,6607	-0,3721	-0,577	0,7128	0,6868	-0,4785	-0,4974	-0,6444	-0,6358	-0,6104	-0,6224
P value (two-tailed)	0,009	0,0089	0,0074	0,0075	0,0148	0,0193	0,2336	0,0495	0,0093	0,0136	0,1156	0,0999	0,0237	0,0263	0,035	0,0307
R squared	0,511	0,5115	0,5288	0,5277	0,4636	0,4366	0,1385	0,3329	0,508	0,4717	0,229	0,2474	0,4152	0,4043	0,3726	0,3874
**Echocardiography 56d and 4h rOSTd**																
Parameter	**PA ESA**	**PA EDA**	**AP ESA**	**AP EDA**	**PA EDD**	**PA ESD**	**PA EDAW**	**PA EDPW**	**AP EDD**	**AP ESD**	**AP EDAW**	**AP EDPW**	**PA 2D FS**	**AP 2D FS**	**PA MM FS**	**AP MM FS**
Number of XY Pairs	12	12	12	12	12	12	12	12	12	12	12	12	12	12	12	12
Pearson r	0,2503	0,2422	0,2019	0,1727	0,2678	0,2544	-0,2031	-0,02382	0,3201	-0,06793	-0,3363	-0,3424	-0,4872	-0,4583	-0,261	-0,3765
P value (two-tailed)	0,4326	0,4482	0,5291	0,5916	0,4	0,4249	0,5267	0,9414	0,3105	0,8339	0,2852	0,2759	0,1082	0,1341	0,4126	0,2278
R squared	0,06267	0,05865	0,04078	0,02981	0,07174	0,06473	0,04124	0,0005676	0,1024	0,004614	0,1131	0,1173	0,2374	0,21	0,06811	0,1417
**Echocardiography 56d and 5h rOSTd**																
Number of XY Pairs	12	12	12	12	12	12	12	12	12	12	12	12	12	12	12	12
Pearson r	0,7603	0,7614	0,7278	0,7354	0,5797	0,6446	-0,6462	-0,4723	0,681	0,4647	-0,5835	-0,6132	-0,5747	-0,5481	-0,5592	-0,2528
P value (two-tailed)	0,0041	0,004	0,0073	0,0064	0,0482	0,0236	0,0232	0,121	0,0148	0,128	0,0464	0,034	0,0506	0,065	0,0587	0,4279
R squared	0,578	0,5797	0,5297	0,5408	0,336	0,4155	0,4176	0,2231	0,4638	0,2159	0,3405	0,376	0,3303	0,3004	0,3127	0,06391
**Echocardiography 56d and 6h rOSTd**																
Number of XY Pairs	12	12	12	12	12	12	12	12	12	12	12	12	12	12	12	12
Pearson r	0,7372	0,7418	0,738	0,7336	0,5122	0,7061	-0,594	-0,265	0,7851	0,2851	-0,5818	-0,6002	-0,6748	-0,6867	-0,6064	-0,4422
P value (two-tailed)	0,0062	0,0057	0,0061	0,0066	0,0887	0,0103	0,0417	0,4052	0,0025	0,369	0,0472	0,0391	0,0161	0,0136	0,0366	0,15
R squared	0,5435	0,5502	0,5447	0,5382	0,2623	0,4985	0,3528	0,07023	0,6163	0,08129	0,3385	0,3603	0,4553	0,4716	0,3677	0,1956

Organs 56d and 4h rOSTd		
Parameter	Lung/BW	LV/BW
Number of XY Pairs	11	11
Pearson r	-0,2475	0,3041
P value (two-tailed)	0,4631	0,3632
R squared	0,06127	0,09248
**Organs 56d and 5h rOSTd**		
Number of XY Pairs	11	11
Pearson r	0,2776	0,7428
P value (two-tailed)	0,4086	0,0088
R squared	0,07704	0,5518
**Organs 56d and 6h rOSTd**		
Number of XY Pairs	11	11
Pearson r	0,4817	0,7429
P value (two-tailed)	0,1336	0,0088
R squared	0,232	0,552

Fig S6. Correlations of rOSTd 4, 5 and 6 hours after MI and relevant long-term parameters (echocardiography, organ/bodyweight ratios) after MI.. Abbreviations: PA: papillary, AP: apical, ESA: endsystolic area, EDA: enddiastolic area, EDD: enddiastolic diameter, ESD: endsystolic diameter, EDAW: enddiastolic anterior wall, EDPW: enddiastolic posterior wall, MM: M-Mode, FS: fractional shortening. rWG: relative weight gain, BW: body weight, LV: left ventricle, r-values lower/higher than -0.5 to 0.5 are marked in yellow, lower/higher than -0.6 to 0.6 are marked in orange, lower/higher than -0.7 to 0.7 are marked in red.
